# Unilateral Unusual Post-traumatic Breast Pain and Swelling in a Young Adult: A Case of Tuberculous Empyema Necessitans

**DOI:** 10.7759/cureus.66914

**Published:** 2024-08-15

**Authors:** Mohamed Khalil Khabet, Hamza Retal, Erika De Smet, Anis Soualili, Redouane Kadi

**Affiliations:** 1 Radiology, Erasmus University Hospital, Brussels, BEL; 2 Radiology, Helora University Hospital, Nivelles, BEL

**Keywords:** empyema necessitans, pleurocutaneous fistula, enhanced chest ct, mycobacterium tuberculosis, empyema thoracis, tuberculous empyema necessitans

## Abstract

Empyema necessitans is a very rare and morbid complication of pleural empyema. It is defined as the extension of pleural infection to the chest wall and surrounding soft tissues. Our case highlights an unusual presentation of empyema necessitans in a 29-year-old man. The patient had no prior comorbidities and presented to the emergency department with a 15-day history of growing left unilateral chest pain and swelling. This was initially clinically misdiagnosed as a post-traumatic hematoma. Contrast-enhanced chest CT scan allowed a diagnosis and the ruling out of the main differentials, such as skeletal lesions extending to adjacent structures but also benign and malignant soft tissue masses. The treatment involved surgical drainage of the abscess. Microbiological analysis of the abscess content identified *Mycobacterium tuberculosis* as the causative pathogen. The patient was subsequently treated with antituberculous drugs, leading to a favorable clinical outcome. This case outlines the importance of an enhanced chest CT scan in making an early diagnosis, defining the extent of the disease, and discussing differentials, all of which are paramount to better results with fewer complications. Moreover, it highlights the fact that blunt trauma may facilitate the formation of a fistula when an underlying infection is present.

## Introduction

Empyema necessitans was first described by Gullan De Baillon in the 17th century when he observed the occurrence of empyema necessitans following the spontaneous rupture of a syphilitic aneurysm. It is an unusual and rare complication where a collection of pus in the pleural space extends beyond the pleural cavity into the chest wall soft tissues and adjacent structures (sinus tract). Empyema necessitans commonly extends into the subcutaneous tissues of the chest wall but can also spread to involve deeper structures such as the esophagus, pericardial and paravertebral regions, peritoneum, and retroperitoneal spaces. It can be very destructive and lead to osteomyelitis, especially of the ribs [1]. The resulting subcutaneous abscess may eventually rupture through the skin, leading to a transparietal pleuro-cutaneous fistula. Most of the time, clinical findings are subtle, which may lead to diagnostic confusion. Our case is meant to remind clinicians and radiologists of the importance of both a thorough clinical examination and adapted imaging techniques in making an accurate and prompt diagnosis to reduce the morbidity of this deadly complication.

## Case presentation

**Figure 1:**
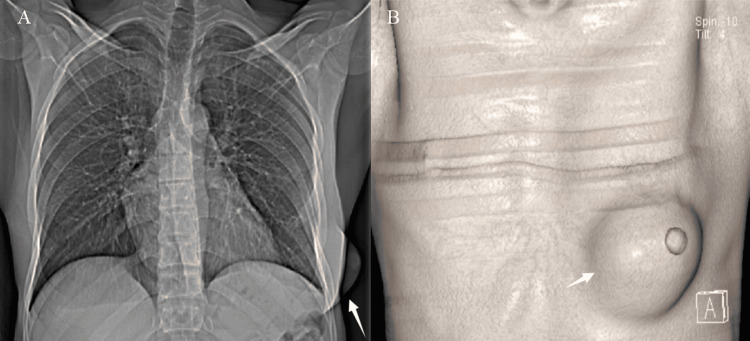
Chest X-ray and CT scan reconstruction demonstrating left breast soft tissue expansion. Image (A) is a posteroanterior (PA) chest X-ray revealing thickening and swelling of the soft tissues of the left thoracic wall with breast asymmetry (white arrow) and a slight diffuse increase in peribronchovascular markings. Image (B) is a chest CT with a 3D reformat demonstrating the significant expansion of the left breast, lifting the nipple (white arrow).

A 29-year-old man living in Morocco who recently arrived in Belgium, with no prior comorbidities, presented to the emergency department with a 15-day history of growing chest pain and swelling. The patient’s medical history, based on queries, was uneventful except for minor trauma to the left chest wall a month before when he fell from his bike that resulted in skin abrasion according to the patient. At physical examination, vitals were stable and the patient presented no fever. An expansion of the left breast lifting the nipple was obvious. It was associated with a loss of skin substance and purulent leakage. No further associated symptoms or abnormalities were noted. Blood sample analyses showed discrete hyperneutrophilia and lymphopenia with moderate inflammatory syndrome. Initial evaluation through a chest X-ray revealed chest wall swelling and breast asymmetry on the left side with little prominence of bronchovascular markings. A 3D reformat of the chest CT that was realized later demonstrated the significant expansion of the left breast, lifting the nipple (arrow), due to the chest wall collection (Figure 1).

**Figure 2:**
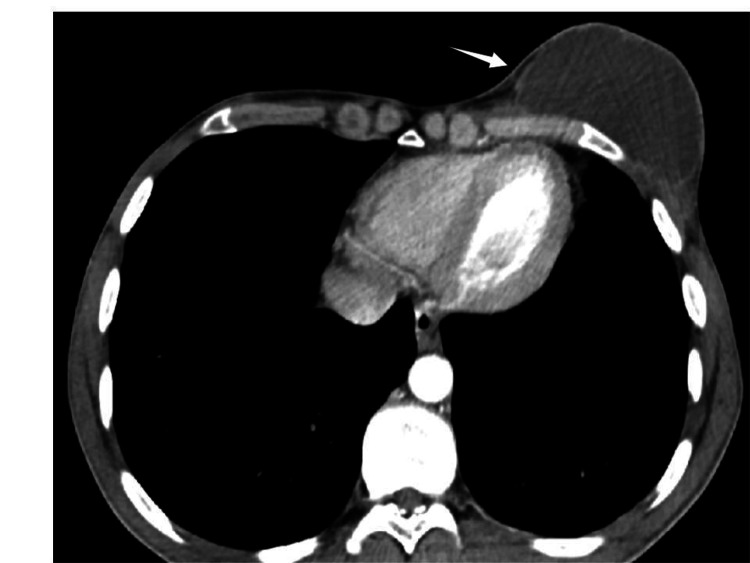
Enhanced axial slice of chest CT in the mediastinal window demonstrating a well-defined hypodense fluid collection with a thin wall and fluid density on the left superior thoracic wall mimicking a breast prosthesis (white arrow).

Based on clinical examination and the post-traumatic context, a diagnosis of an infected post-traumatic chest hematoma through skin abrasion was proposed. An enhanced chest CT was ordered to confirm the diagnosis and exclude differentials. It revealed a well-defined and thin-walled hypodense collection with fluid density (15 HU) and discrete peripheral enhancement on the left superior thoracic wall at the level of the fourth intercostal space, mimicking a breast prosthesis (Figure 2). 

**Figure 3:**
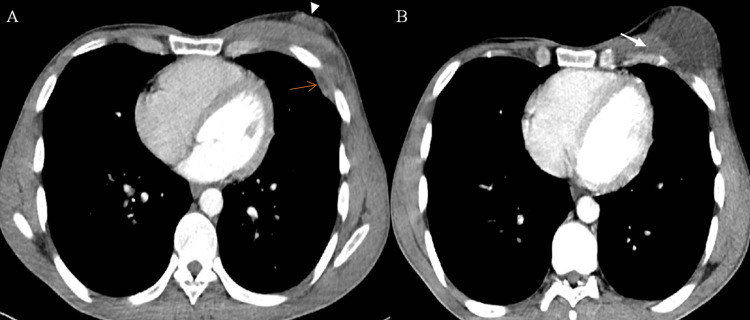
Enhanced axial slice of chest CT in the mediastinal window demonstrating the chest wall collection and the empyema. Image (A) demonstrates a deep communication between the chest wall collection and a slight heterogeneous pleural empyema (orange arrow) along with a chest wall defect in the chest wall communicating with the same collection, indicating the presence of a superficial fistula (arrowhead). Image (B) highlights a breast tissue infiltration and thickening of the pectoralis major muscle (white arrow).

A deep communication “fistula” with pleural heterogeneous empyema was noted along with a discrete defect in the chest wall communicating with the collection, indicating the presence of a superficial fistula in association with a reactional infiltration of the breast tissue up to a thickened pectoralis major muscle (Figure 3).

**Figure 4:**
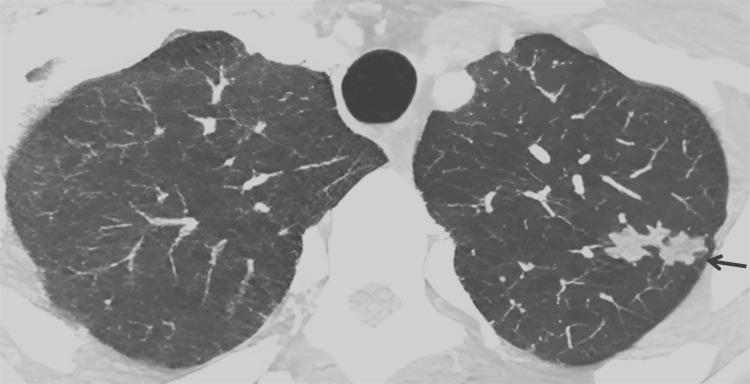
Enhanced axial slice of chest CT in the lung window with the maximum intensity projection (MIP) showing a tree-in-bud appearance in the left upper lobe (black arrow), highly suggestive of endobronchial dissemination of infectious diseases.

There were no signs of rib fracture or osteitis/osteomyelitis. Vascular structures were normal with no evidence of venous thrombosis. The images obtained at the level of the left upper lobe showed on the lung window multiple areas of centrilobular nodules with a linear branching pattern giving a tree-in-bud appearance, which is very suggestive of endobronchial dissemination of infectious diseases (Figure 4).

The subcutaneous collection was surgically drained, and research of pathogens in the pus including tuberculosis culture medium allowed the isolation of *Mycobacterium tuberculosis* (MT). Here, we remind that TB culture has a very low sensitivity in such cases. The final diagnosis of pulmonary tuberculosis with empyema necessitans and pleuro-cutaneous fistula was retained.

## Discussion

Necessitans in Latin means necessary. Even if the extension of empyema thoracis is not necessary and frequent as it may seem from the meaning of the word, it is the expected evolution of a poorly treated/uncontrolled purulent pleural or pulmonary intraparenchymal collection. The “primum movens” in empyema necessitans is an exudative parapneumonic effusion due to local irritation secondarily infected through a mechanism of germ translocation or fistula formation. A build-up of pressure ensues with an increase in bacterial count (or any other infectious agent), fibrin, and debris inside the pleural cavity that leads necessarily to the extension of the disease to adjacent structures, especially to the thoracic wall soft tissues through what one may call “least resistance pathways." This dramatic evolution is essentially seen due to the aggressiveness of the infectious agent implicated or poorly treated/uncontrolled pneumonia/pulmonary abscess. Immunosuppression and a history of thoracic surgery must be sought in all cases [2]. Bronchial stenting was also reported as a risk factor for empyema necessitans [3].

In our case, this was provoked by the evolution of insidious unknown pulmonary tuberculosis in an otherwise healthy 29-year-old male coming from an endemic country to tuberculosis. The patient had no recent contact with confirmed or suspected pulmonary TB cases. He had neither a surgical record nor an immunosuppression. The chest wall collection evolved in an unusually confusing post-traumatic context. The incidence of empyema necessitans has drastically declined due to the use of antibiotics. MT remains the most common causative infectious agent, representing nearly 70% of cases found in the literature, followed by methicillin-resistant *Staphylococcus aureus* (MRSA) [1]. A few empyema necessitans cases have also been reported in the literature due to infection with *Actinomyces* species, *Aspergillus fumigatus* [4], *Nocardia farcinica* [5], and *Streptococcus pneumoniae*. Keeping in mind these epidemiological parameters, both radiologists and clinicians should alert microbiologists to the possibility of a specific germ such as MT to ease its identification using Ziehl-Neelsen staining and an appropriate growth medium. This has been done in our case, which prompted relatively quick isolation of MT via the appropriate growth medium [6].

The radiological appearance on chest X-ray is often nonspecific and may demonstrate soft tissue swelling associated with consolidation and/or pleural effusion or it can appear normal. In some cases, as in ours, a soft tissue density in the chest wall can be seen. An enhanced chest CT scan remains the gold standard for making an accurate diagnosis. It allows a precise assessment of the extension both through the chest wall to soft tissues and deep involvement of the mediastinum and skeletal structures. As with any other deep and extensive infection, venous thrombosis must be considered as a potential complication and should be excluded. Herein, we emphasize the importance of CT assessment for possible complications, such as broncho-pleural fistula [7] resulting usually from a spontaneous rupture of empyema in the pulmonary bronchi [8], which may lead to mediastinal compression, and mediastinitis as possible complications in addition to peritoneal extension. To improve the efficacy and safety of aspirations, ultrasound should be used, especially for small and relatively deep collections. In our case, an ultrasound was not performed.

The differential diagnosis of a chest wall collection/mass includes hematoma/seroma, soft tissue infection with abscess and skeletal lesions but also benign and malignant soft tissue tumors. Among malignant neoplasms, invasive ductal carcinoma is a top differential especially in women as it remains rare in males. Mesothelioma, sarcomas, bronchogenic carcinoma, and primary pulmonary neoplasms can also be observed. However, neoplasms in general will have a different clinical context and more solid components. Infectious endocarditis and transdiaphragmatic spread of abdominal infections should be kept in mind. Although they are rare, noninfectious etiologies should also be considered, such as Wegener’s granulomatosis and sarcoidosis [5].

**Figure 5:**
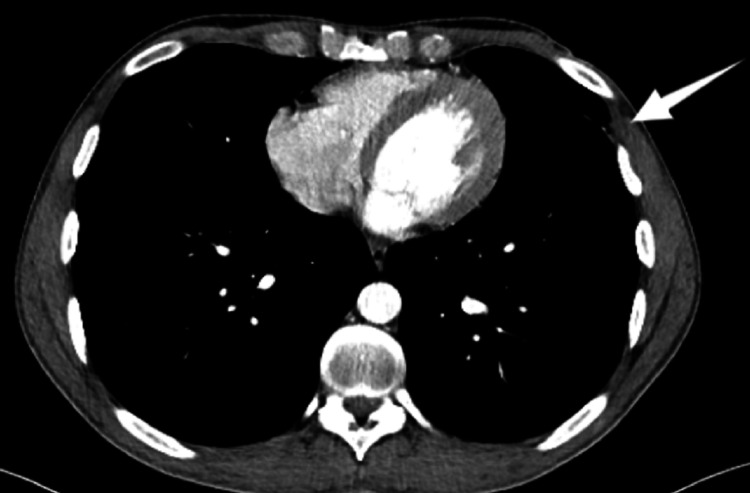
Enhanced axial slice of the chest CT in the mediastinal window at three months post-treatment demonstrating complete resolution of the chest wall collection and the presence of a pleural cicatricial band at the site of the previously observed fistula (arrow).

In our case, the previous trauma on the chest wall was likely the initial event in the course of the disease, allowing a secondary infection to graft at the injured site. The infection then spread into the adjacent subcutaneous tissue, crossing over the chest wall towards the left breast, where it formed a subcutaneous abscess with a transparietal pleuro-cutaneous fistula. The patient being from an endemic country for tuberculosis, the clinical context and the radiological pattern led us to suspect the tuberculous origin of empyema necessitans, which was later confirmed microbiologically. Our patient was successfully treated by simple surgical drainage without the need for reconstruction. The surgical approach is not yet standardized due to the scarcity of cases. Analgesics and antituberculous drug therapy (ethambutol/isoniazid/pyrazinamide/rifampicin) were started. After showing improvement, the patient was discharged and encouraged to take his antituberculous medication for the next six months with periodic follow-up. Three months later, the chest wound was healing, and an enhanced chest CT scan showed a complete regression of the pleural empyema and fistula which were replaced by a pleural fibrotic band (Figure 5).

## Conclusions

Empyema necessitans remains a rare disease defined as the extension of empyema thoracis to adjacent structures, especially to the chest wall. It should be kept in mind due to its morbidity and possible fatal evolution if unrecognized or poorly treated. Osteomyelitis and venous thrombosis are among the most common complications. Blunt trauma may facilitate the formation of a fistula and the extension of the disease. Enhanced chest CT remains the gold standard in making an early and accurate diagnosis of EN, excluding differentials, and screening for complications, granting adapted therapy and lesser morbidity. After all, prevention must be the primary goal in this setting and is achieved by efficient detection and treatment of thoracic infections.

## References

[1] Tripathi S, Meena DS, Rohila AK (2021). Empyema necessitans with osteomyelitis of fifth rib due to Nocardia farcinica: a case report. BMC Infect Dis.

[2] Almeida Borges J, Madama D (2023). Empyema necessitans: after recent thoracostomy in an immunocompromised patient. Respirol Case Rep.

[3] Moezinia CJ, Choo HM, Chreif H, Madani Y (2023). Empyema necessitans secondary to bronchial stenting. Thorax.

[4] Benjanuwattra J, Leelaviwat N, Guerin C, Patel PU, Mekraksakit P, Nugent K (2022). Empyema necessitans as a rare manifestation of Aspergillus fumigatus infection. Proc (Bayl Univ Med Cent).

[5] Ishikawa K, Mori N (2022). Empyema necessitans due to Nocardia farcinica. IDCases.

[6] Acar M, Sutcu M, Akturk HG (2016). A case of empyema necessitatis in a child with Mycobacterium tuberculosis. Clin Pediatr (Phila).

[7] Risal R, Jahir T, Islam R (2022). A rare case of empyema complicated with bronchopleural fistula secondary to mucormycosis in a young immunocompromised diabetic patient with COVID-19. Cureus.

[8] Crouch A, Lay J, Neeki A, Dong F, Neeki M (2021). Spontaneous rupture of empyema necessitans in the emergency department. Cureus.

